# Association of thyroid antibodies status on the outcomes of pregnant women with hypothyroidism (maternal hypothyroidism on pregnancy outcomes, MHPO-4)

**DOI:** 10.1186/s12884-021-03594-y

**Published:** 2021-02-15

**Authors:** Zareen Kiran, Aisha Sheikh, Najmul Islam

**Affiliations:** Section of Endocrinology, Department of Medicine, The Aga Khan University, P.O. Box 3500, Stadium Road, Karachi, Pakistan

**Keywords:** Thyroid disorders, Thyroid antibodies, Pregnancy, Complications, Outcomes

## Abstract

**Background:**

Autoimmunity increases with age and is often commonly evaluated in women of the reproductive age group. Prevalence of thyroid antibodies is common even in euthyroid pregnant women. We aim to compare the association of thyroid antibody status on the maternal and neonatal outcomes in pregnant women with hypothyroidism.

**Methods:**

We conducted a cross-sectional retrospective study on 718 cases in the Aga Khan University Hospital. Information was collected on pregnant women who have been diagnosed with hypothyroidism before conception or during their antenatal period. Laboratory data were recorded for thyroid peroxidase antibodies, anti-thyroglobulin antibodies, and thyroid-stimulating hormone levels. Maternal and neonatal outcomes were also noted from medical file records. Data analysis was performed on Statistical Package for the Social Sciences version 20.0.

**Results:**

Overall, 146 out 718 cases were included for final analysis. Thyroid peroxidase antibodies were positive in 66.4% and anti-thyroglobulin was positive in 52.1% cases, whereas 43.8% of cases had both antibodies positive. Pre-gestational diabetes was significantly associated with thyroid autoimmunity. There was a 73% less chance of gestational hypertension for thyroid autoimmune groups. Gestational diabetes and maternal (chronic) hypertension were found to have an independent effect on postpartum hemorrhage. Hypertensive disorders in pregnancy were found to have an independent risk for premature birth.

**Conclusion:**

Our study reports a 74.7% prevalence of positive thyroid antibodies in hypothyroid pregnant women, with higher association with pre-gestational diabetes. Gestational hypertension was least likely to occur in thyroid autoimmune groups. None of the outcomes were independently associated with worse outcomes.

**Supplementary Information:**

The online version contains supplementary material available at 10.1186/s12884-021-03594-y.

## Background

Pregnancy is a state of changing metabolic as well as hormonal physiology. Most commonly, several changes occur in thyroid functions during pregnancy. Although autoimmunity in pregnancy is supposed to be less frequent than in non-pregnant state because of immune suppression, thyroid antibodies are relatively common during pregnancy even with normal thyroid functions with varying prevalence of 5 to 15% [1–4]. Any failure to adapt to gestational physiological changes results in thyroid dysfunction which poses a risk to maternal and fetal outcomes, although there are universal controversies [5–7]. Studies have shown that thyroid antibodies can adversely affect pregnancy outcomes even in euthyroid women [8]. These antibodies have also been suggested to have an independent association with adverse pregnancy outcomes [9].

The exact pathophysiology for these adverse effects is not known but it has been speculated that presence of anti-thyroid antibodies is part of a generalized autoimmune imbalance that may be responsible for increased complications [10]. Women who are positive for thyroid antibodies before pregnancy may develop subclinical hypothyroidism, possibly due to latent thyroid dysfunction which becomes overt due to the increasing demands during pregnancy [11–13]. Moreover, the presence of anti-thyroid antibodies is associated with infertility and such women get pregnant at a delayed age placing them in a generally high-risk category and increased fetal loss [14, 15]. One study has suggested an increased risk of gestational diabetes with anti-thyroid antibodies as compared to antibody-negative women [16]. On the other hand, antibody status does not affect the outcome of postpartum hemorrhage for either subclinical hypothyroidism or euthyroid women in a study with healthy controls [17]. It is therefore imperative to assess the risk of these outcomes in the individual population.

Very few studies have been reported from Pakistan regarding the prevalence of thyroid antibodies status [18] and outcomes of antibody-positive pregnant women [19]. But, none of them have compared the presence or absence of antibody status on the maternal and neonatal outcomes of pregnancy. Therefore, we aim to compare the outcomes of hypothyroid pregnant women to their anti-thyroid antibody status for the first time from Pakistan.

## Methods

### Study design

We conducted a retrospective chart review of hypothyroid pregnant patients from their preconception through the complete course of gestation, from 2008 till 2016, presenting to Aga Khan University, Karachi, Pakistan.

### Study setting

The hypothyroid pregnant females presenting to endocrine and obstetric clinics at the Aga Khan University Hospital.

### Sample size assumption

The sample size is calculated using Open Source Epidemiologic Statistics for Public Health, (Open Epi) version 3 [20]. The maternal outcomes of pregnancies affected by hypothyroidism, from the literature showed that spontaneous abortions were 17% [8], in patients with thyroid peroxidase antibodies positive (Anti-TPO +) women. Therefore, taking the frequency of 17% as an outcome, with a 95% confidence level and a bound on the error of ±6% the estimated sample size was 244 pregnant women.

### Sampling technique

Non-probability consecutive sampling. Purposive sampling was employed.

### Eligibility criteria

#### Inclusion criteria

We selected patients, according to the following inclusion criteria:

- Hypothyroid pregnant women on levothyroxine replacement with doses adjusted appropriately to aim controlled hypothyroidism with TSH ≤2. 5 mIU/L throughout pregnancy [4].
- Neonates of these hypothyroid pregnant women.

#### Exclusion criteria

Hypothyroid pregnant women whose antibodies were not checked were excluded from the study.

### Data collection

We conducted a retrospective review of the medical record files of pregnant women, during the years 2008–2016. We selected these women by applying coding words of hypothyroidism and pregnancy in the electronic medical record maintained, according to the international classification of diseases (ICD) coding system in the Health Information and Management System (HIMS) department of our hospital. Data were collected by trained medical doctors. It was randomly double-checked and corroborated by the principal investigator. We noted the age, parity, BMI, and comorbidities of these pregnant women. Data of outcomes like live births, pregnancy loss (including miscarriage, stillbirth/intrauterine death, medical termination of pregnancy, and ectopic pregnancy), gestational hypertension, pre-eclampsia, postpartum hemorrhage, placental abruption, and mode of delivery were noted for all pregnancies from obstetric discharge summaries. Neonatal outcomes including hyperbilirubinemia, hypocalcemia, preterm birth, birth weight, neonatal intensive care admission (NICU), APGAR (Appearance, Pulse, Grimace, Activity, and Respiration) score, and neonatal death were recorded from respective neonatal file records.

#### Thyroid stimulating hormone levels and thyroid antibodies

TSH levels available before conception and during each month throughout pregnancy were noted. TSH levels were assessed by Advia Centaur (Siemens Diagnostics), Chemiluminescence immunoassay. The functional sensitivity limit of the assay is 0.008 μIU/mL, with the assay detection range spanning from 0.008–150 μIU/mL to detect the lowest and highest abnormal value. The normal laboratory reference range of TSH for adults is 0.4–4.2 μIU/mL for the age group 21–54 years in our hospital. Thyroid peroxidase antibodies (anti-TPO) and thyroglobulin antibodies (anti-TG) were assayed on Immulite 2000 (Siemens Diagnostics) using the Chemiluminescence immunoassay technique. The reference range of anti-TPO antibodies for normal healthy people was less than 35 IU/ML, whereas for anti-TG antibodies it was less than 40 IU/ml.

#### Definitions of maternal and neonatal outcomes

We defined maternal outcomes as per internationally accepted criteria.

- Pregnancy loss: (1) *Spontaneous Abortion*; defined as loss of a pregnancy at or before 20 weeks of gestation. (2) *Intrauterine death (IUD) or stillbirth*; defined as a pregnancy loss occurring after 20 weeks of gestation [21, 22]. Other outcomes include ‘*Medical termination of pregnancy*’ and ‘*Ectopic pregnancy*’ as determined by the clinician based on obstetric examination and investigations.
- Gestational Hypertension (GH): Women who started on anti-hypertensives during antenatal visits were labeled as GH, a condition characterized by high blood pressure that develops after week 20 in pregnancy [23]. Others were labeled as ‘Chronic Hypertension’.
- Pre-eclampsia: Pregnant women characterized by both high blood pressure (> 140/90 mmHg) and proteinuria (≥ 0.3 g/24 h) that develops after 20 weeks of gestation in a previously normotensive woman [24].
- Postpartum Hemorrhage: Defined as a blood loss of 500 ml or more within 24 h after birth [25].
- Placental Abruption: Defined as the separation of the placenta from uterine lining any time after the 20th week of pregnancy.
- Modes of delivery: These were noted as emergency or elective cesarean sections, spontaneous vaginal delivery with or without episiotomy, and finally instrumental delivery.
- Term: We divided the gestational age at birth into ‘extremely preterm (<28)’, ‘very preterm (28-31)’, ‘late preterm (32-36)’, ‘term (37-42)’ and ‘post-term (> 42)’.
- Birth Weight: Defined as ‘Small for Gestation Age; SGA (<2500g)’, ‘Appropriate for Gestational Age; AGA (2500-4000g)’ and ‘Large for Gestational Age; LGA (>4000g)’.
- Neonatal jaundice: Total bilirubin levels above 1 mg/dL checked within 48–72 h of birth.
- Low APGAR score at 5 min: We noted the one minute APGAR score (scored from zero to ten) as a means of assessing the baby’s physical health and then the five-minute score for assessing how the baby has responded to any resuscitation attempts. A score below 7 is considered low at 5 min.
- NICU admission: Neonates requiring ICU admission for 24 h or more.

### Statistical analysis

Descriptive analysis was performed for demographic and clinical features and results are presented as number (percentage). We grouped BMI into underweight (< 18.5), normal (18.5–24.9), overweight (25–29.9), and obese (> = 30) categories. Age was categorized into different age ranges. Similarly, parity was also categorized into different groups. Preconception TSH was categorized into levothyroxine adequately replaced (TSH ≤2.5 μIU/mL) and inadequately replaced (TSH > 2.5 μIU/mL) groups. Medians of TSH were calculated for each trimester and then categorized into similar groups. We calculated the distribution antibody status among these two groups. Univariate analysis of maternal and neonatal outcomes of thyroid antibody-positive hypothyroid pregnancies was assessed using the Chi-square and Fischer Exact test. Statistical significance was taken as *P*-value ≤0.05. Odds ratios and their 95% confidence intervals (95% CI) were used to quantify the extent of the association of antibodies with dichotomous outcomes. To find out the association of thyroid antibodies with maternal and neonatal outcomes, we stepwise performed multivariate logistic regression analysis for each outcome concerning the baseline maternal factors and calculated the adjusted odds ratio. Only those outcomes which had a statistically significant association with maternal characteristics are described. Data is analyzed using Statistical Package for the Social Sciences version 20.

## Results

**Fig. 1:**
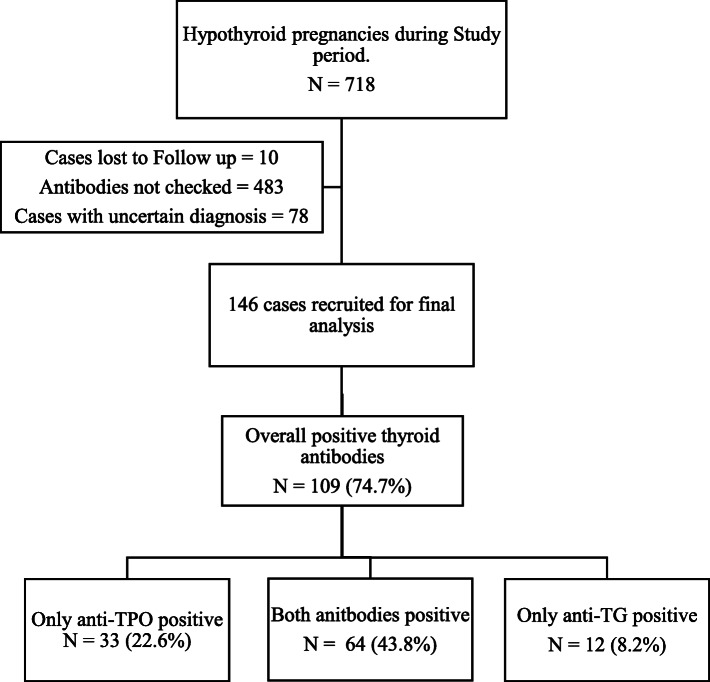
Flow chart showing selection of our study population and antibody status

Figure 1 describes our patient’s selection for this study. A total of 718 cases were retrieved using hypothyroidism and pregnancy code from the department of HIMS. Ten cases were lost to follow up, and only 225 cases were found to have thyroid antibodies checked. Out of these 225 cases, 78 cases had an uncertain diagnosis, so finally 146 cases were included for our analysis. In our study, the prevalence of overall thyroid antibody positivity was 74.7% (*n* = 109). Individually, only anti-TPO was positive in 22.6% (*n* = 33) of all cases and anti-TG in 8.2% (*n* = 12) cases. Almost 44% (*n* = 64) cases had both antibodies positive. Out of 146 cases, 116 cases were diagnosed before pregnancy, and their antibodies were checked pre-pregnancy. The remaining 30 cases had their antibodies checked at different times during pregnancy (Supplementary file 1). In those with thyroid antibodies negative, 33 cases had an unknown etiology for primary hypothyroidism, whereas, 2 had postsurgical hypothyroidism and one had post-radioactive iodine ablation hypothyroidism and the other had hypothyroidism following subacute thyroiditis. Maternal characteristics and outcomes of the whole cohort of 718 patients had been reported in a separate paper (MHPO-1) [26]. Medians of TSH determined before conception as well as in each trimester for this study are 3.17 μIU/mL (69.9%), 3.83 μIU/mL (67.8%), 2.51 μIU/mL (74.7%) and 2.13 μIU/mL (72.6%). Distribution of anti-TPO and anti- TG antibodies among adequately and inadequately replaced levothyroxine cases for each trimester is presented in Supplementary file 2.

Table 1 summarizes the association of thyroid antibody status with baseline characteristics of hypothyroid pregnancies. The majority of the women were in the age group of 26–30 (41.8%). None of the maternal characteristics were significantly associated with thyroid antibody status. However, amongst the comorbid conditions, only pre-gestational diabetes mellitus (pre- GDM) was found to have a strong association with anti-TPO antibodies (*p*-value 0.018).

**Table 1:** Association of thyroid antibodies with baseline characters in hypothyroid pregnancies

	Anti-TPO		***P*** value	Anti-TG		***P*** value
	Positive N (%) *N* = 97	Negative N (%) *N* = 49		Positive N (%) *N* = 76	Negative N (%) *N* = 70	
**Age Groups**						
Age 20–25	8 (8.2)	8 (16.3)		8 (10.5)	8 (11.4)	
Age 26–30	41 (42.3)	20 (40.8)	0.243	27 (35.5)	34 (48.6)	0.363
Age 31–35	28 (28.9)	16 (32.7)		27 (35.5)	17 (24.3)	
Age 36 and above	20 (20.6)	5 (10.2)		14 (18.4)	11 (15.7)	
**Parity**^a^						
Nulliparous	30 (31.9)	20 (42.6)	0.213	22 (29.7)	28 (41.8)	0.135
Multiparous	64 (68.1)	27 (57.4)		52 (70.3)	39 (58.2)	
**BMI**						
Underweight (< 18.5)	4 (4.7)	2 (4.7)		3 (4.6)	3 (4.7)	
Normal (18.5–24.9)	33 (38.4)	13 (30.2)		25 (38.5)	21 (32.8)	
Overweight (25–29.9)	30 (34.9)	15 (34.9)	0.826^b^	25 (38.5)	20 (31.2)	0.565^b^
Obesity class I (30–34.9)	16 (18.6)	11 (25.6)		10 (15.4)	17 (26.6)	
Obesity class II and above (> = 35)	3 (3.5)	2 (4.7)		2 (3.1)	3 (4.7)	
**Pre-Gestational Diabetes**						
Yes	2 (2.1)	6 (12.2)	**0.018**^b^	2 (2.7)	6 (8.6)	0.155^b^
No	94 (97.9)	43 (87.8)		73 (97.3)	64 (91.4)	
**Gestational Diabetes**						
Yes	21 (21.9)	5 (10.2)	0.083	12 (16.0)	14 (20.0)	0.530
No	75 (78.1)	44 (89.8)		63 (84.0)	56 (80.0)	
**Hypertension**						
No	84 (87.5)	38 (77.6)		65 (86..7)	57 (81.4)	
Gestational Hypertension/Pre-eclampsia	6 (6.2)	7 (14.3)	0.248^b^	4 (5.3)	9 (12.9)	0.250^b^
Chronic Hypertension	6 (6.2)	4 (8.2)		6 (8.0)	4 (5.7)	

*Anti-TPO *anti-thyroid peroxidase antibodies,* Anti-TG *anti-thyroglobulin antibodies*, BMI* Body mass index. *P*-values calculated by Chi-square test applied. ^a^Data of 5 cases are missing. ^b^Fischer exact test applied

### Association of thyroid antibodies with maternal outcomes

The odds ratio of thyroid antibodies with maternal outcomes is presented in Table 2. Compared to mothers with both antibodies negative, there was a 73% less chance of gestational hypertension with an unadjusted odds ratio being 0.26 (*p*-value 0.005) for anti-TPO and 0.27 (p-value 0.011) for anti-TG. This remained significant only for anti-TG antibodies after adjusting for maternal age, BMI, DM, and GDM (Table 3). However, maternal parity was an independent risk factor for gestational hypertension [adjusted OR 0.20 (p-value 0.05) for anti-TPO and 0.20 (p-value 0.04) for anti-TG]. Out of other maternal risk factors, GDM and maternal (chronic) hypertension was found to have an independent effect on postpartum hemorrhage in thyroid autoimmune groups upon multivariate logistic regression (Table 4). But none of the other maternal outcomes had any significant relationship with thyroid antibody status, and there was no difference in maternal outcomes of anti-TPO and anti-TG positive or negative groups upon adjusting for other maternal risk factors.

**Table 2:** Association of thyroid antibodies with maternal outcomes in hypothyroid pregnancies

Maternal Outcomes	Anti-TPO		***P*** value	Odds ratio	95% CI	Anti-TG		***P*** value	Odds ratio	95% CI
	Positive N (%) N = 97	Negative N (%) N = 49				Positive N (%) N = 76	Negative N (%) N = 70			
**Abortions**										
Yes	8 (8.2%)	4 (8.2%)	1.000	1.011	0.289-	6 (7.9%)	6 (8.6%)	0.915	1.059	0.371-
No	89 (91.8%)	45 (91.8%)			3.539	70 (92.1%)	64 (91.4%)			3.026
**Gestational hypertension**										
Yes	7 (4.9%)	13 (16.0%)	0.005	0.267	0.102-	5 (4.3%)	15 (13.9%)	0.011	0.277	0.097-
No	137 (95.1%)	68 (84.0%)			0.701	112 (95.7%)	93 (86.1%)			0.790
**Pre-eclampsia**										
Yes	8 (5.6%)	4 (4.9%)	1.000^a^	1.132	0.330–3.883	7 (6.0%)	5 (4.6%)	0.652	1.311	0.403–4.261
No	136 (94.4%)	77 (95.1%)				110 (94.0%)	103 (95.4%)			
**Postpartum Hemorrhage**										
Yes	51 (35.4%)	37 (45.7%)	0.130	0.652	0.374–1.136	43 (36.8%)	45 (41.7%)	0.450	0.814	0.476-
No	93 (64.6%)	44 (54.3%)				74 (63.2%)	63 (58.3%)			1.391
**Mode of labor**										
Spontaneous	29 (22.8%)	10 (13.3%)	0.207	1.923^b^	0.878-	17 (16.5%)	22 (22.2%)	0.136	0.692^b^	0.342-
Induced	27 (21.3%)	24 (32.0%)			4.213	24 (23.3%)	27 (27.3%)			1.398
Augmented	20 (15.7%)	10 (13.3%)				21 (20.4%)	9 (9.1%)			
Not applicable due to CS	51 (40.2%)	31 (41.3%)				41 (39.8%)	41 (41.4%)			
**Mode of delivery**										
Em-LSCS	28 (22.0%)	24 (32.0%)	0.649	0.838^b^	0.465-	(18.4%)	33 (33.3%)	0.072	0.763^b^	0.432-
LSCS	48 (37.8%)	24 (32.0%)			1.512	41 (39.8%)	31 (31.3%)			1.347
Instrumental	6 (4.7%)	3 (4.0%)				6 (5.8%)	3 (3.0%)			
SVD	17 (13.4%)	9 (12.0%)				11 (10.7%)	15 (15.2%)			
SVD with episiotomy	28 (22.0%)	15 (20.0%)				26 (25.2%)	17 (17.2%)			

*Em-LSCS* emergency lower segment caesarean section, *SVD* spontaneous vaginal delivery. *P*-values calculated by Chi square test applied. ^a^Fischer test applied. *P*-value significant at <=0.05. ^b^Odds ratio for spontaneous mode of labor and for C-section mode of delivery

**Table 3:** Association of independent variables with gestational hypertension adjusted for anti-thyroid peroxidase (Anti-TPO) and anti-thyroglobulin (Anti-TG) antibodies on multivariate logistic regression

	Unadjusted OR (95% CI)	***P***-value	Adjusted OR (95% CI)	***P***-value	Unadjusted OR (95% CI)	***P***-value	Adjusted OR (95% CI)	***P***-value
**ANTI-THYROID PEROXIDASE**					**ANTI-THYROGLOBULIN**			
**Yes**	1	0.095	1	0.08	1	0.047	1	0.04*
**No**	2.50 (0.85–7.38)		2.17 (0.92–7.26)		3.35 (1.01–11.08)		3.12 (1.73–4.53)	
**AGE GROUPS**								
**Age 20–25**	1				1			
**Age 26–30**	0.45 (0.26–1.45)	0.34			0.45 (0.26–1.45)	0.34		
**Age 31–35**	0.25 (0.15–1.33)				0.25 (0.15–1.33)			
**Age 36 and above**	0.36 (0.12–1.56)				0.36 (0.12–1.56)			
**PARITY**								
**Nulliparous**	1		1		1		1	
**Primiparous**	0.67 (0.20–2.23)	0.04	0.78 (0.44–0.96)	0.05*	0.67 (0.20–2.23)	0.04	0.84 (0.54–0.96)	0.04*
**Biparous/multiparous**	0.11 (0.012–0.95)		0.20 (0.12–1.02)		0.11 (0.012–0.95)		0.20 (0.1–1.3)	
**BODY MASS INDEX**								
**Underweight/Normal**	1	0.194			1	0.194		
**Overweight/obese**	2.39 (0.64–8.89)				2.39 (0.64–8.89)			
**PRE GESTATIONAL DIABETES**								
**Yes**	3.17 (0.58–17.39)	0.182			3.17 (0.58–17.39)	0.182		
**No**	1				1			
**GESTATIONAL DIABETES**								
**Yes**	0.3 (0.03–2.38)	0.25			0.3 (0.03–2.38)	0.25		
**No**	1				1			

*Fischer test applied. *P*-value significant at <=0.05

**Table 4:** Association of independent variables with postpartum hemorrhage adjusted for anti-thyroid peroxidase (Anti-TPO) and anti-thyroglobulin (Anti-TG) antibodies on multivariate logistic regression

	Unadjusted OR (95% CI)	***P***-value	Adjusted OR (95% CI)	***P***-value	Unadjusted OR (95% CI)	***P***-value	Adjusted OR (95% CI)	***P***-value
**ANTI-THYROID PEROXIDASE**					**ANTI-THYROGLOBULIN**			
**Positive**	1		1		1		1	
**Negative**	1.46 (0.72–2.99)	0.29	1.56 (0.35–1.92)	0.21	1.36 (0.68–2.69)	0.88	1.45 (0.83–3.16)	0.74
**AGE GROUPS**								
**Age 20–25**	1				1			
**Age 26–30**	1.07 (0.32–3.5)	0.11			1.07 (0.32–3.5)	0.11		
**Age 31–35**	0.85 (0.23–2.87)				0.85 (0.23–2.87)			
**Age 36 and above**	2.8(0.74–10.47)				2.8(0.74–10.47)			
**PARITY**								
**Nulliparous**	1				1			
**Primiparous**	0.68 (0.38–1.62)	0.60			0.68 (0.38–1.62)	0.60		
**Biparous/multiparous**	1.01 (0.44–2.29)				1.01 (0.44–2.29)			
**BODY MASS INDEX**								
**Underweight/Normal**	1	0.253			1	0.253		
**Overweight/obese**	1.53 (0.73–3.17)				1.53 (0.73–3.17)			
**PRE GESTATIONAL DIABETES**								
**Yes**	1.91 (0.45–8.02)	0.373			1.91 (0.45–8.02)	0.373		
**No**	1				1			
**GESTATIONAL DIABETES**								
**Yes**	3.84 (1.64–9.28)	0.03	3.35 (2.61–4.21)	0.01*	3.84 (1.64–9.28)	0.03	2.38 (1.54–3.45)	0.012*
**No**	1		1		1		1	
**HYPERTENSION**								
**No**	1		1		1		1	
**Pre-eclampsia/ Gestational Hypertension**	2.68 (1.32–4.07)	0.01	2.82 (2.02–4.16)	0.04*	2.68 (1.32–4.07)	0.01	3.01 (2.14–4.27)	0.029*
**Chronic Hypertension**	5.36 (1.31–21.87)		3.21 (1.59–4.56)		5.36 (1.31–21.87)		3.21 (1.59–4.56)	

*Fischer test applied. *P*-value significant at <=0.05

### Association of thyroid antibodies with neonatal outcomes

Table 5 presents the association of thyroid antibodies with neonatal outcomes. Neonates of our hypothyroid mothers had a 76.8% less chance of prolonged NICU stay in those with anti-TG positive status with an odds ratio (95% CI) of 0.232 (0.062–0.878) (*p*-value 0.022). Similarly, neonatal respiratory distress syndrome (NRDS) in a neonate is 87.3% less predictable in the anti-TG positive group with an odds ratio (95% CI) of 0.127 (0.015–1.064) (p-value 0.033). However, these outcomes had no significant association upon adjusting for other maternal factors. Hypertensive disorders in pregnancy were the only maternal factor found to have an independent risk for premature birth in antibody-positive groups upon multivariate logistic regression (Table 6).

**Table 5:** Association of thyroid antibodies with neonatal outcomes in hypothyroid pregnancies

	Anti-TPO		P value	Odds ratio	95% CI	Anti-TG		P value	Odds ratio	95% CI
Neonatal outcomes	Positive N (%) *N* = 83	Negative N (%) *N* = 44				Positive N (%) *N* = 64	Negative N (%) *N* = 63			
**Low APGAR at 5 min**										
Yes	1 (1.2%)	0 (0.0%)	1.000^a^	1.524	1.343-	0 (0.0%)	1 (1.6%)		2.049	1.712-
No	82 (98.8%)	43 (100.0%)			1.731	64 (100.0%)	61 (98.4%)	0.492^a^		2.452
**Premature Birth**										
Yes	18 (21.7%)	13 (29.5%)	0.593	0.660	0.287–1.517	13 (20.3%)	18 (28.6%)	0.307	0.637	0.281-
No	65 (78.3%)	31 (70.5%)				51 (79.7%)	45 (71.4%)			1.444
**Low birth weight**										
Yes	18 (22.0%)	10 (22.7%)	0.920	0.956	0.398–2.300	10 (15.6%)	18 (29.0%)	0.070	0.453	0.190-
No	64 (78.0%)	34 (77.3%)				54 (84.4%)	44 (71.0%)			1.080
**Neonatal Respiratory distress**										
Yes	5 (6.0%)	3 (6.8%)	0.861	0.876	0.199-	1 (1.6%)	7 (11.1%)	0.033^a^	0.127	0.015-
No	78 (94.0%)	41 (93.2%)			3.850	63 (98.4%)	56 (88.9%)			1.064
**Sepsis**										
Yes	2 (2.4%)	2 (4.5%)	0.609^a^	0.519	0.071-	1 (1.6%)	3 (4.8%)	0.302	0.317	0.032-
No	81 (97.6%)	42 (95.5%)			3.813	63 (98.4%)	60 (95.2%)			3.137
**Neonatal Jaundice**										
Yes	32 (38.6%)	12 (27.3%)	0.204	1.673	0.754-	23 (35.9%)	21 (33.3%)	0.758	1.122	0.540-
No	51 (61.4%)	32 (72.7%)			3.713	41 (64.1%)	42 (66.7%)			2.332
**Hypocalcaemia**										
Yes	4 (4.8%)	3 (6.8%)	0.693^a^	0.692	0.148-	2 (3.1%)	5 (7.9%)	0.235	0.374	0.070-
No	79 (95.2%)	41 (93.2%)			3.240	62 (96.9%)	58 (92.1%)			2.005
**NICU admission < 24 h**										
Yes	0 (0.0%)	0 (0.0%)	–	–	–	0 (0.0%)	0 (0.0%)	–	–	–
No	83 (100.0%)	44 (100.0%)				64 (100.0%)	63 (100.0%)			
**NICU admission > 24 h**										
Yes	10 (12.0%)	4 (9.1%)	0.613	1.370	0.404–4.649	3 (4.7%)	11 (17.5%)	0.022	0.232	0.062–0.878
No	73 (88.0%)	40 (90.9%)				61 (95.3%)	52 (82.5%)			
**Neonatal death**										
Yes (Early & Late)	2 (2.4%)	0 (0.0%)	0.544^a^	1.543	1.356-	2 (3.1%)	0 (0.0%)	0.496^a^	2.016	1.690-
No	81 (97.6%)	44 (100.0%)			1.756	62 (96.9%)	63 (100.0%)			2.406
**Gestational age**										
Preterm (28–36)	21 (25.3%)	13 (29.5%)	0.607	1.238^b^	0.548-	16 (25.0%)	18 (28.6%)	0.649	1.200	0.546-
Term (37–42)	62 (74.7%)	31 (70.5%)			2.797	48 (75.0%)	45 (71.4%)			2.635
**Birth weight**										
Small for Gestational Age (SGA <2500)	19 (23.2%)	10 (22.7%)	0.955	1.025	0.429-	11 (17.2%)	18 (29.0%)	0.114	0.507	0.217-
Appropriate for Gestational Age (AGA 2500–4000)	63 (76.8%)	34 (77.3%)			2.452	53 (82.8%)	44 (71.0%)			1.187

*P*-values calculated by Chi square test applied. ^a^Fischer exact test applied. APGAR, Appearance, Pulse, Grimace, Activity, and Respiration. NICU, neonatal intensive care admission. ^b^Odds ratio for Preterm

**Table 6:** Association of independent variables with premature birth adjusted for anti-thyroid peroxidase (Anti-TPO) and anti-thyroglobulin (Anti-TG) antibodies on multivariate logistic regression

	Unadjusted OR (95% CI)	*P*-value	Adjusted OR (95% CI)	*P*-value	Unadjusted OR (95% CI)	*P*-value	Adjusted OR (95% CI)	*P*-value
**ANTI-THYROID PEROXIDASE**					**ANTI-THYROGLOBULIN**			
**Yes**	1	0.328	1	0.58	1	0.32	1	0.46
**No**	1.51 (0.65–3.47)		1.39 (0.57–3.39)		1.56 (0.69–3.55)		1.44 (0.43–3.64)	
**AGE GROUPS**								
**Age 20–25**	1				1			
**Age 26–30**	3.124 (0.82–11.77)	0.19			3.124 (0.82–11.77)	0.19		
**Age 31–35**	2.5 (0.87–2.43)				2.5 (0.87–2.43)			
**Age 36 and above**	2.25 (0.67–7.53)				2.25 (0.67–7.53)			
**PARITY**								
**Nulliparous**	1				1			
**Primiparous**	0.48(0.17–1.36)	0.30			0.48(0.17–1.36)	0.30		
**Biparous/mutiparous**	0.54 (0.20–1.45)				0.54 (0.20–1.45)			
**BODY MASS INDEX**								
**Underweight/Normal**	1	0.529			1	0.529		
**Overweight/obese**	1.31 (0.55–3.10)				1.31 (0.55–3.10)			
**PRE GESTATIONAL DIABETES**								
**Yes**	1.24 (0.22–6.74)	0.80			1.24 (0.22–6.74)	0.80		
**No**	1				1			
**GESTATIONAL DIABETES**								
**Yes**	2.35 (0.93–5.92)	0.07			2.35 (0.93–5.92)	0.07		
**No**	1				1			
**HYPERTENSION**								
**No**	1		1		1		1	
**Pre-eclampsia/ Gestational Hypertension**	2. 65(1.81–8.63)	0.002	2.48 (0.72–8.53)	0.03*	2. 65(1.81–8.63)	0.002	2.46(0.83–8.77)	0.05*
**Chronic Hypertension**	12.74 (2.34–36.91)		12.71 (2.37–67.99)		12.74 (2.34–36.91)		11.34 (2.63–64.34)	

*Fischer test applied.* P*-value significant at <=0.05

## Discussion

Our study found overall 66.4% of the women to be anti-TPO positive and 52.1% to be anti-TG positive amongst hypothyroid pregnant cohort (including overlapping and alone positive cases for each antibody, ref. Fig. 1). We compared 6 maternal and 12 distinct neonatal outcomes for thyroid antibody status as prior studies demonstrated an associated risk with several thyroid autoimmune disorders. Our women were on levothyroxine replacement and the only significant difference between adequately and inadequately replaced levothyroxine cases was found during the second trimester (*p*-value 0.007). Only pre-gestational diabetes was significantly associated with anti-TPO antibodies in our study. Autoimmunity was associated with less chance of gestational hypertension, less prolonged stay in NICU for neonates and lesser possibility of developing NRDS. However, hypertensive disorders of pregnancy and GDM were found to have an independent risk of postpartum hemorrhage, and only hypertensive disorders are associated with an independent risk of premature birth in our study.

In an earlier study from our center, a 13.5% prevalence of anti-TPO was reported in a population of 943 subjects [18]. The presence of thyroid anti-TPO antibodies was found positive in 40% of hypothyroid pregnant females in an Indian epidemiological study [27], while an Iranian study reported 12.8 and 8.5% prevalence of anti-TPO and anti-TG antibody positivity in their population of 600 pregnant females [28]. More differences in data are reported even from the same region in the literature, as anti-TPO was positive in 27.8% of pregnant women in another study from India [29]. These differences could be related to iodine intake in the general population [30] and the timing of antibody evaluation during gestation, as the autoimmunity progressively declines [31, 32]. Thus, the lack of universal recommendations about screening strategies due to insufficient evidence by the endocrinologists and obstetricians has resulted in confusing reports about the antibody prevalence and pregnancy outcome risks [33].

The relationship between type 1 diabetes and thyroid autoimmunity is well established in the literature [34, 35]. On the other hand, the occurrence of GDM in the thyroid autoimmune group is also evident in several studies [36, 37]. Very few studies are available to describe the relation between thyroid antibodies and pre-GDM. Our study reported a significant association of anti-TPO antibodies in the pre-GDM group which is different from that reported in earlier studies by Shahbazian et al. [38] and Konar et al. [39] and even in a recent report from Ghamri et al. [40].

According to the literature, a higher rate of miscarriages is associated with anti-TPO positive cases [41–44], but several studies report no significant effect on the incidence of abortion or other adverse pregnancy outcomes due to raised thyroid antibodies, supporting our findings [45–47].

Moncef Feki et al. reported that women with positive anti-TPO have a higher prevalence of gestational hypertension and other adverse pregnancy outcomes [48]. However, in a prospective Indian study on 1000 women, no differences were observed for the occurrence of any hypertensive disorders of pregnancy along with several other outcomes [15]. In this study an interesting association of gestational hypertension with an anti-TPO antibody has been found, where, more women with normal blood pressure had antibody positivity even before pregnancy, as reported in a systematic review by Nazarpour et al. [49]. This is somewhat also similar to a case-control study by Alvi A. et al. in which there were more antibody-positive cases in the normotensive or control group (*p*-value = 0.007) [50]. Our study showed that the presence of GDM and hypertension had an independent risk of postpartum hemorrhage, even after adjusting for thyroid antibody status and other maternal risk factors, as reported recently [51, 52].

Neonates of hypothyroid mothers are generally reported to have prolonged NICU stay than euthyroid women [53, 54]. In two large studies, by Negro et al. and Meena A. et al. there was no significant association between thyroid antibody positivity and NICU admission [55, 56], but they had analyzed only anti-TPO antibodies in their study designs. Our analysis showed a significant chance of less prolonged NICU stay with negative anti-TG antibodies which is unusual and never reported before. However, there was no risk of prolonged ICU stay in anti-TPO positive groups (OR 1.370, 95% CI, 0.404–4.649, *p*-value 0.613).

Literature is scarce about the association of maternal thyroid disorders and neonatal respiratory distress syndrome, nevertheless, with controversial views [54, 57]. The effect of thyroid autoimmunity is even more rarely reported for this particular outcome. A study from Negro et al. reported a greater association of NRDS (*p*-value 0.005) with anti-TPO antibody positivity [55]. However, our study reported a rare finding of a lesser chance of NRDS in those with anti-TG antibodies. We need to further analyze these findings in large prospective studies. Furthermore, the presence of hypertensive disorders in pregnancy had an independent risk of premature birth in our neonates, as reported in the literature [58]. Moreover, a similar finding was published decades ago in the hypothyroid pregnant population [59].

There are several limitations to our study, the most important being that, the results of this study cannot be generalized to all pregnant populations as screening for thyroid dysfunction or autoimmunity is not universally performed. This study, however, can be regarded as a baseline reflection of our hypothyroid pregnant population. Moreover, this is the only study with a large number of patients from a tertiary hospital in Pakistan.

## Conclusion

Our study reports a 74.7% prevalence of positive thyroid antibodies in hypothyroid pregnant women. These antibodies had a higher association with diabetes mellitus. With our research, we propose that normal blood pressure rather than gestational hypertension, either before or during pregnancy was significantly associated with thyroid autoimmunity. The positive antibody was associated with less prolonged NICU admission and less neonatal respiratory distress syndrome in the neonates of hypothyroid mothers.

## Supplementary Information

**Additional file 1.**

**Additional file 2.**

## Data Availability

The dataset supporting the findings of this study can be made available upon request to the first author whose email is drzareenkiran@gmail.com.

## Abbreviations

TSH: Thyroid Stimulating Hormone
FT4: Free Thyroxine
TPO: Thyroid peroxidase
PIH: pregnancy-induced hypertension
C-section: Caesarean section
LSCS: Lower segment cesarean section
PPH: Postpartum hemorrhage

## References

[1] Allan W, Haddow J, Palomaki G, Williams J, Mitchell M, Hermos R (2000). Maternal thyroid deficiency and pregnancy complications: implications for population screening. J Med Screen.

[2] Casey BM, Dashe JS, Wells CE, McIntire DD, Byrd W, Leveno KJ (2005). Subclinical hypothyroidism and pregnancy outcomes. Obstet Gynecol.

[3] van den Boogaard E, Vissenberg R, Land JA, van Wely M, van der Post JA, Goddijn M (2011). Significance of (sub) clinical thyroid dysfunction and thyroid autoimmunity before conception and in early pregnancy: a systematic review. Hum Reprod Update.

[4] De Groot L, Abalovich M, Alexander EK, Amino N, Barbour L, Cobin RH (2012). Management of thyroid dysfunction during pregnancy and postpartum: an Endocrine Society clinical practice guideline. J Clin Endocrinol Metab..

[5] Glinoer D (1997). The regulation of thyroid function in pregnancy: pathways of endocrine adaptation from physiology to pathology. Endocr Rev.

[6] Glinoer D, Riahi M, Grün J-P, Kinthaert J (1994). Risk of subclinical hypothyroidism in pregnant women with asymptomatic autoimmune thyroid disorders. J Clin Endocrinol Metab.

[7] Negro R, Formoso G, Mangieri T, Pezzarossa A, Dazzi D, Hassan H (2006). Levothyroxine treatment in euthyroid pregnant women with autoimmune thyroid disease: effects on obstetrical complications. J Clin Endocrinol Metab..

[8] Stagnaro-Green A, Roman SH, Cobin RH, El-Harazy E, Alvarez-Marfany M, Davies TF (1990). Detection of at-risk pregnancy by means of highly sensitive assays for thyroid autoantibodies. Jama..

[9] Nor Azlin M, Bakin Y, Mustafa N, Wahab N, Johari M, Kamarudin N (2010). Thyroid autoantibodies and associated complications during pregnancy. J Obstet Gynaecol.

[10] Kroopnick JM, Kim CS (2016). Overview of hypothyroidism in pregnancy. Semin Reprod Med.

[11] Yoshioka W, Amino N, Ide A, Kang S, Kudo T, Nishihara E (2015). Thyroxine treatment may be useful for subclinical hypothyroidism in patients with female infertility. Endocr J.

[12] Stagnaro-Green A, Dogo-Isonaige E, Pearce EN, Spencer C, Gaba ND (2015). Marginal iodine status and high rate of subclinical hypothyroidism in Washington DC women planning conception. Thyroid.

[13] Bliddal S, Boas M, Hilsted L, Friis-Hansen L, Tabor A, Feldt-Rasmussen U (2015). Thyroid function and autoimmunity in Danish pregnant women after an iodine fortification program and associations with obstetric outcomes. Eur J Endocrinol.

[14] Seungdamrong A (2016). The impact and Management of Subclinical Hypothyroidism for improving reproductive outcomes such as fertility and miscarriage. Semin Reprod Med.

[15] Meena M, Chopra S, Jain V, Aggarwal N (2016). The effect of anti-thyroid peroxidase antibodies on pregnancy outcomes in Euthyroid women. J Clin Diagnostic Res.

[16] Ying H, Tang YP, Bao YR, Su XJ, Cai X, Li YH (2016). Maternal TSH level and TPOAb status in early pregnancy and their relationship to the risk of gestational diabetes mellitus. Endocrine..

[17] Gur EB, Karadeniz M, Inceefe H, Tatar S, Turan G, Genc M (2015). Thyroid antibodies in euthyroid and subclinical hypothyroidic pregnant women with autoimmune hypothyroidism: effects on hematological parameters and postpartum hemorrhage. Ginekol Pol.

[18] Iqbal S, Ghani F, Qureshi R (2016). Frequency of thyroid peroxidase antibody and its association with miscarriages among pregnant women. J Coll Phys Surg Pak.

[19] Ghafoor F, Mansoor M, Malik T, Malik MS, Khan AU, Edwards R (2006). Role of thyroid peroxidase antibodies in the outcome of pregnancy. J Coll Phys Surg Pak.

[20] Dean A, Sullivan KM, Soe MM (2013). OpenEpi: open source epidemiologic statistics for public health, version.

[21] Organization WH. Maternal, newborn, child and adolescent health: Adolescent development. URL http://www.who.int/maternal_child_adolescent/topics/adolescence/dev/en. 2015.

[22] Da Silva FT, Gonik B, McMillan M, Keech C, Dellicour S, Bhange S (2016). Stillbirth: case definition and guidelines for data collection, analysis, and presentation of maternal immunization safety data. Vaccine..

[23] Davey DA, MacGillivray I (1988). The classification and definition of the hypertensive disorders of pregnancy. Am J Obstet Gynecol.

[24] Sibai BM (2003). Diagnosis and management of gestational hypertension and preeclampsia. Obstet Gynecol.

[25] Organization WH (2014). WHO recommendations for the prevention and treatment of postpartum haemorrhage. 2012.

[26] Kiran Z, Sheikh A, Malik S, Meraj A, Masood M, Ismail S (2019). Maternal characteristics and outcomes affected by hypothyroidism during pregnancy (maternal hypothyroidism on pregnancy outcomes, MHPO-1). BMC Pregnancy Childbirth.

[27] Dhanwal DK, Bajaj S, Rajput R, Subramaniam K, Chowdhury S, Bhandari R (2016). Prevalence of hypothyroidism in pregnancy: an epidemiological study from 11 cities in 9 states of India. Indian J Endocrinol Metab..

[28] Saki F, Dabbaghmanesh MH, Ghaemi SZ, Forouhari S, Omrani GR, Bakhshayeshkaram M (2015). Thyroid autoimmunity in pregnancy and its influences on maternal and fetal outcome in Iran (a prospective study). Endocr Res.

[29] Rajput R, Goel V, Nanda S, Rajput M, Seth S (2015). Prevalence of thyroid dysfunction among women during the first trimester of pregnancy at a tertiary care hospital in Haryana. Indian J Endocrinol Metab.

[30] Chen X, Jin B, Xia J, Tao X, Huang X, Sun L, et al. Effects of thyroid peroxidase antibody on maternal and neonatal outcomes in pregnant women in an iodine-sufficient area in China. Int J Endocrinol. 2016;2016: Article ID 6461380, 8 pages. 10.1155/2016/6461380. 10.1155/2016/6461380 PMC4738937 26884759

[31] Amino N, Kuro R, Tanizawa O, Tanaka F, Hayashi C, Kotani K (1978). Changes of serum anti-thyroid antibodies during and after pregnancy in autoimmune thyroid diseases. Clin Exp Immunol.

[32] Ekinci EI, Chiu WL, Lu ZX, Sikaris K, Churilov L, Bittar I (2015). A longitudinal study of thyroid autoantibodies in pregnancy: the importance of test timing. Clin Endocrinol.

[33] Ahmed IZ, Eid YM, El Orabi H, Ibrahim HR (2014). Comparison of universal and targeted screening for thyroid dysfunction in pregnant Egyptian women. Eur J Endocrinol.

[34] Kawasaki E (2014). Type 1 diabetes and autoimmunity. Clin Pediatr Endocrinol.

[35] Michels AW, Gottlieb PA (2010). Autoimmune polyglandular syndromes. Nat Rev Endocrinol.

[36] Sharmeen M, Shamsunnahar PA, Laita TR, Chowdhury SB (2014). Overt and subclinical hypothyroidism among Bangladeshi pregnant women and its effect on fetomaternal outcome. Bangladesh Med Res Counc Bull.

[37] Negro R, Stagnaro-Green A (2014). Diagnosis and management of subclinical hypothyroidism in pregnancy. Bmj..

[38] Shahbazian H, Shahbazian N, Baniani MR, Yazdanpanah L, Latifi SM (2013). Evaluation of thyroid dysfunction in pregnant women with gestational and pre-gestational diabetes. Pak J Med Sci.

[39] Konar H, Sarkar M, Roy M (2018). Association of Thyroid Dysfunction and Autoimmunity in pregnant women with diabetes mellitus. J Obstetrics Gynaecol India.

[40] Ghamri KA, Ghamri RA (2020). Evaluation of thyroid dysfunction and thyroid antibodies among subjects with gestational and pre-gestational diabetes at king Abdulaziz University hospital, Jeddah: a retrospective analysis (2014-2018). Int J Pharm Res Allied Sci.

[41] Iijima T, Tada H, Hidaka Y, Mitsuda N, Murata Y, Amino N (1997). Effects of autoantibodies on the course of pregnancy and fetal growth. Obstet Gynecol.

[42] Dendrinos S, Papasteriades C, Tarassi K, Christodoulakos G, Prasinos G, Creatsas G (2000). Thyroid autoimmunity in patients with recurrent spontaneous miscarriages. Gynecol Endocrinol.

[43] Glinoer D, Soto MF, Bourdoux P, Lejeune B, Delange F, Lemone M (1991). Pregnancy in patients with mild thyroid abnormalities: maternal and neonatal repercussions. J Clin Endocrinol Metab..

[44] Lejeune B, Grun JP, De Nayer P, Servais G, Glinoer D (1993). Antithyroid antibodies underlying thyroid abnormalities and miscarriage or pregnancy induced hypertension. BJOG Int J Obstet Gynaecol.

[45] Blumenthal NJ, Eastman CJ (2017). Beneficial effects on pregnancy outcomes of thyroid hormone replacement for subclinical hypothyroidism. J Thyroid Res.

[46] Tong Z, Xiaowen Z, Baomin C, Aihua L, Yingying Z, Weiping T (2016). The effect of subclinical maternal thyroid dysfunction and autoimmunity on intrauterine growth restriction: a systematic review and meta-analysis. Medicine..

[47] Plowden TC, Schisterman EF, Sjaarda LA, Zarek SM, Perkins NJ, Silver R (2016). Subclinical hypothyroidism and thyroid autoimmunity are not associated with fecundity, pregnancy loss, or live birth. J Clin Endocrinol Metab..

[48] Feki M, Omar S, Menif O, Tanfous NB, Slimane H, Zouari F (2008). Thyroid disorders in pregnancy: frequency and association with selected diseases and obstetrical complications in Tunisian women. Clin Biochem.

[49] Nazarpour S, Ramezani Tehrani F, Simbar M, Azizi F (2015). Thyroid dysfunction and pregnancy outcomes. Iran J Reprod Med.

[50] Alavi A, Adabi K, Nekuie S, Jahromi EK, Solati M, Sobhani A (2012). Thyroid dysfunction and autoantibodies association with hypertensive disorders during pregnancy. J Pregnancy.

[51] Muche AA, Olayemi OO, Gete YK (2020). Effects of gestational diabetes mellitus on risk of adverse maternal outcomes: a prospective cohort study in Northwest Ethiopia. BMC Pregnancy Childbirth.

[52] Durmaz A, Komurcu N (2018). Relationship between maternal characteristics and postpartum hemorrhage: a meta-analysis study. J Nurs Res.

[53] Mannisto T, Mendola P, Grewal J, Xie Y, Chen Z, Laughon SK (2013). Thyroid diseases and adverse pregnancy outcomes in a contemporary US cohort. J Clin Endocrinol Metab.

[54] Mannisto T, Mendola P, Reddy U, Laughon SK (2013). Neonatal outcomes and birth weight in pregnancies complicated by maternal thyroid disease. Am J Epidemiol.

[55] Negro R, Schwartz A, Gismondi R, Tinelli A, Mangieri T, Stagnaro-Green A (2011). Thyroid antibody positivity in the first trimester of pregnancy is associated with negative pregnancy outcomes. J Clin Endocrinol Metab.

[56] Meena A, Nagar P (2016). Pregnancy outcome in euthyroid women with anti-thyroid peroxidase antibodies. J Obstetrics Gynecol India.

[57] Spencer L, Bubner T, Bain E, Middleton P (2015). Screening and subsequent management for thyroid dysfunction pre-pregnancy and during pregnancy for improving maternal and infant health. Cochrane Database Syst Rev.

[58] Premkumar A, Baer RJ, Jelliffe-Pawlowski LL, Norton ME (2019). Hypertensive disorders of pregnancy and preterm birth rates among black women. Am J Perinatol.

[59] Leung AS, Millar LK, Koonings PP, Montoro M, Mestman JH (1993). Perinatal outcome in hypothyroid pregnancies. Obstet Gynecol.

