# Supplementary material for: Association of thyroid antibodies status on the outcomes of pregnant women with hypothyroidism (maternal hypothyroidism on pregnancy outcomes, MHPO-4)
Source: BMC Pregnancy Childbirth. 2021 Feb 15;21:136. doi: 10.1186/s12884-021-03594-y (PMC7885223; doi:10.1186/s12884-021-03594-y)
Supplement: Supplementary file 1 — Additional file 1. [file 12884_2021_3594_MOESM1_ESM.rtf]

S1: Timings of antibody tests in hypothyroid pregnant women.  

Cases diagnosed during pregnancy 
N = 30 (20.5%)*	Cases diagnosed prior to pregnancy 
N = 116 (79.5%)*	
1st trimester 
2nd trimester  
3rd trimester 	4 (2.7)		
	9 (6.2)		
	4 (2.7)		
*Data of 13 cases are missing. 
 
