# Supplementary material for: Association of thyroid antibodies status on the outcomes of pregnant women with hypothyroidism (maternal hypothyroidism on pregnancy outcomes, MHPO-4)
Source: BMC Pregnancy Childbirth. 2021 Feb 15;21:136. doi: 10.1186/s12884-021-03594-y (PMC7885223; doi:10.1186/s12884-021-03594-y)
Supplement: Supplementary file 2 — Additional file 2. [file 12884_2021_3594_MOESM2_ESM.rtf]

Table 1: Distribution of Anti-TPO antibodies in hypothyroid women adequately versus inadequately replaced with levothyroxine during 1st trimester. 

PREGNANT COHORT IN 1ST TRIMESTER  	Anti-TPO	*P value	
	positive	negative		
Hypothyroid women on adequate levothyroxine replacement (TSH up to 2.5)	21
67.7%	10
32.3%	.279	
Hypothyroid women on inadequate levothyroxine replacement (TSH 2.6 and above)	53
77.9%	15
22.1%		
*Pearson Chi-Square applied

Table 2: Distribution of Anti-TG antibodies in hypothyroid women adequately versus inadequately replaced with levothyroxine during 1st trimester. 


PREGNANT COHORT IN 1ST TRIMESTER  	Anti-TG	*P value	
	positive	negative		
Hypothyroid women on adequate levothyroxine replacement (TSH up to 2.5)	16
51.6%	15
48.4%	.502	
Hypothyroid women on inadequate levothyroxine replacement (TSH 2.6 and above)	40
58.8%	28
41.2%		
*Pearson Chi-Square applied

Table 3: Distribution of Anti-TPO antibodies in hypothyroid women adequately versus inadequately replaced with levothyroxine during 2nd trimester. 

PREGNANT COHORT IN 2nd TRIMESTER  	Anti-TPO	*P value	
	positive	negative		
Hypothyroid women on adequate levothyroxine replacement (TSH up to 2.5)	43
78.2%	12
21.8%	.007	
Hypothyroid women on inadequate levothyroxine replacement (TSH 2.6 and above)	29
53.7%	25
46.3%		
*Pearson Chi-Square applied


Table 4: Distribution of Anti-TG antibodies in hypothyroid women adequately versus inadequately replaced with levothyroxine during 2nd trimester. 

PREGNANT COHORT IN 2nd TRIMESTER  	Anti-TG	*P value	
	positive	negative		
Hypothyroid women on adequate levothyroxine replacement (TSH up to 2.5)	29
52.7%	26
47.3%	.633	
Hypothyroid women on inadequate levothyroxine replacement (TSH 2.6 and above)	26
48.1%	28
51.9%		
*Pearson Chi-Square applied


Table 5: Distribution of Anti-TPO antibodies in hypothyroid women adequately versus inadequately replaced with levothyroxine during 3rd trimester. 

PREGNANT COHORT IN 3rd TRIMESTER  	Anti-TPO	*P value	
	positive	negative		
Hypothyroid women on adequate levothyroxine replacement (TSH up to 2.5)	41
65.1%	22
34.9%	.997	
Hypothyroid women on inadequate levothyroxine replacement (TSH 2.6 and above)	28
65.1%	15
34.9%		
*Pearson Chi-Square applied


Table 6: Distribution of Anti-TG antibodies in hypothyroid women adequately versus inadequately replaced with levothyroxine during 3rd trimester. 

PREGNANT COHORT IN 3rd TRIMESTER  	Anti-TG	*P value	
	positive	negative		
Hypothyroid women on adequate levothyroxine replacement (TSH up to 2.5)	31
49.2%	32
50.8%	.665	
Hypothyroid women on inadequate levothyroxine replacement (TSH 2.6 and above)	23
53.5%	20
46.5%		
*Pearson Chi-Square applied


Table 7: Distribution of Anti-TPO antibodies in hypothyroid women adequately versus inadequately replaced with levothyroxine during Preconception stage.   

PREGNANT COHORT IN PRECONCEPTION STAGE	Anti-TPO	*P value	
	positive	negative		
Hypothyroid women on adequate levothyroxine replacement (TSH up to 2.5)	34
75.6%	11
24.4%	.545	
Hypothyroid women on inadequate levothyroxine replacement (TSH 2.6 and above)	40
70.2%	17
29.8%		
*Pearson Chi-Square applied

Table 8: Distribution of Anti-TG antibodies in hypothyroid women adequately versus inadequately replaced with levothyroxine during Preconception stage.   

PREGNANT COHORT IN PRECONCEPTION STAGE	Anti-TG	*P value	
	positive	negative		
Hypothyroid women on adequate levothyroxine replacement (TSH up to 2.5)	27
60.0%	18
40.0%	.457	
Hypothyroid women on inadequate levothyroxine replacement (TSH 2.6 and above)	30
52.6%	27
47.4%		
*Pearson Chi-Square applied
